# Stereo‐Divergent Enzyme Cascades to Convert Racemic 4‐Phenyl‐2‐Butanol into either (*S*)‐ or (*R*)‐Corresponding Chiral Amine

**DOI:** 10.1002/cbic.202200108

**Published:** 2022-03-03

**Authors:** Maria Romero‐Fernandez, Francesca Paradisi

**Affiliations:** ^1^ School of Chemistry University of Nottingham University Park NG7 2RD Nottingham UK; ^2^ Department of Chemistry Biochemistry and Pharmaceutical Sciences University of Bern Freiestrasse 3 3012 Bern Switzerland

**Keywords:** 4-phenylbutan-2-amine, alcohol amination, biocatalysis, chiral amines, racemic 4-phenyl-2-butanol

## Abstract

The synthesis of enantiopure chiral amines from racemic alcohols is a key transformation in the chemical industry, e. g., in the production of active pharmaceutical ingredients (APIs). However, this reaction remains challenging. In this work, we propose a one‐pot enzymatic cascade for the direct conversion of a racemic alcohol into either (*S*)‐ or (*R*)‐enantiomers of the corresponding amine, with *in‐situ* cofactor recycling. This enzymatic cascade consists of two enantio‐complementary alcohol dehydrogenases, both NADH and NADPH oxidase for *in‐situ* recycling of NAD(P)^+^ cofactors, and either (*S*)‐ or (*R*)‐enantioselective transaminase. This cell‐free biocatalytic system has been successfully applied to the conversion of racemic 4‐phenyl‐2‐butanol into the high value (*S*)‐ or (*R*)‐enantiomers of the amine reaching good (73 % (*S*)) and excellent (>99 % (*R*)) enantioselectivities.

## Graphical abstract

**A one‐pot enzymatic** cascade has been developed for the cell‐free conversion of racemic secondary alcohols to either (*S*)‐ or (*R*)‐enantiopure chiral amines, with *in‐situ* recycling of NAD(P)^+^ cofactors. This biocatalytic system was successfully applied to the amination reaction of racemic 4‐phenyl‐2‐butanol to yield the high value (*S*)‐ or (*R*)‐enantiomers of the amine reaching good (73 % (*S*)) and excellent (>99 % (*R*)) enantioselectivities.

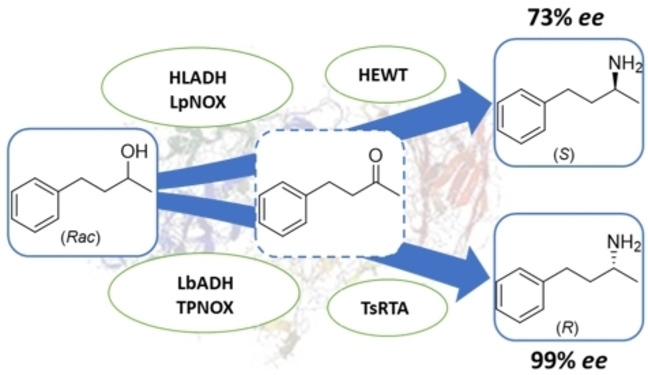

Chiral amines are essential building blocks for the pharmaceutical industry,^[1]^ as they are key intermediates in the synthesis of a plethora of active pharmaceutical ingredients (APIs).^[2]^ The industrial synthesis of chiral amines is mainly developed through metal‐catalysed processes, which mostly require transition metal complexes.^[3]^

During the last decades, biocatalysis emerged as a prominent sustainable alternative to metal‐catalysis in organic chemical synthesis, and nowadays, the enantioselective synthesis of chiral amines by isolated enzymes encompasses a central role in industrial biocatalysis.^[2]^ The first examples of application of isolated enzymes to the production of chiral amines were developed via kinetic resolution of racemic amines by lipases^[4]^ and dynamic kinetic resolution.[^5^, ^6^, ^7^, ^8^] In addition, the direct synthesis of enantiopure amines from prochiral ketones catalysed by enzymes has also been developed, mainly using amine dehydrogenases (AmDHs) and ω‐transaminases (TAs). The former enzymes catalyse the reductive amination of prochiral ketones into chiral amines with the addition of the cofactor NAD(P)H and ammonia, thus producing water as the only by‐product when the cofactor is regenerated in the reaction.[^3^, ^9^, ^10^, ^11^, ^12^] TAs catalyse the transamination of prochiral ketones by using an excess of an amine donor, usually isopropylamine.[^2^, ^13^, ^14^, ^15^, ^16^, ^17^, ^18^] Although several equivalents of sacrificial amino donors are needed to drive the equilibrium to product formation, the use of TAs provides the additional benefit of producing either the (*S*)‐ or the (*R*)‐ enantiomers of the amine, as both (*S*)‐ and (*R*)‐enantioselective TAs have been described.^[19]^ Moreover, several engineered TAs have already proved to be excellent catalysts for the production of complex enantiopure amines.[^19^, ^20^, ^21^]

In contrast to the asymmetric synthesis of amines from ketone precursors, the direct amination of alcohols offers advantages, since the alcohol is generally more easily accessible.^[22]^ In fact, different metal‐catalysed approaches have been proposed to this end.^[23]^ In this context, the biocatalytic amination of alcohols to synthesize chiral amines using enzymatic cascades have also been reported. These reactions involve a two‐step process: alcohol oxidation to form a carbonyl group, followed by reductive amination to produce a primary amine. The use of alcohol dehydrogenase (ADH) for alcohol oxidation and AmDH for the reductive amination step is attractive, since, in principle, the only by‐product is water, and established the hydrogen‐borrowing concept. Several examples of these redox‐neutral enzymatic cascades have been carried out for the asymmetric amination of alcohols.[^3^, ^10^, ^11^, ^24^] On the other hand, TAs have also been successfully applied in the amination step of cascade reactions to synthesize chiral amines. Remarkably, with this strategy, the production of both enantiomers of a broad range of chiral amines has been achieved.[^25^, ^26^, ^27^, ^28^, ^29^, ^30^]

The conversion of easily available racemic alcohols to enantiopure chiral amines would be of course ideal. However, this reaction remains challenging in the pharmaceutical industry as the oxidation of both alcohol enantiomers is required in the first step.^[28]^ Different methodologies have been proposed for the non‐enantioselective oxidation of the alcohol,[^27^, ^28^, ^29^, ^30^] however, the use of two enantio‐complementary ADHs in a biocatalytic approach is appealing.[^25^, ^26^] ADH‐mediated oxidation of alcohols would require stoichiometric amounts of NAD(P)^+^ cofactors, unless this is regenerated *in‐situ* to ensure the sustainability of the process. In cascades involving ADHs‐TAs for the asymmetric synthesis of amines from racemic alcohols, the use of water‐forming NAD(P)H oxidases (NOX) is an interesting approach. This strategy only requires oxygen and generates water as the only by‐product, and it has been shown to enhance the alcohol oxidation step in these enzyme systems.[^25^, ^28^, ^31^]

In this work, we have developed a one‐pot enzyme cascade for the asymmetric synthesis of one aromatic amine from the corresponding racemic secondary alcohol. The system involves two enantio‐complementary ADHs with broad substrate scope, NOX for the *in‐situ* recycling of NAD(P)^+^ cofactor, and either (*S*)‐ or (*R*)‐selective TA. The potential of this enzyme cascade was investigated, adopting as a case study the conversion of the racemic alcohol 4‐phenyl‐2‐butanol into the high value (*S*)‐ and (*R*)‐enantiopure aromatic amines, since the cell‐free enzymatic synthesis of both enantiomers of this amine in a tunable fashion has not been satisfactory. The optimized enzyme cascade provides an alternative for the biosynthesis of chiral amines from racemic alcohols, and to put steps forward in the applicability of biocatalysis in the pharmaceutical industry.

The (*S*)‐selective horse liver alcohol dehydrogenase (HLADH) has been identified for this work as one of the two enantio‐complementary ADHs. Since the 1980s, HLADH has been successfully applied to redox reactions showing excellent enantioselective properties.[^32^, ^33^, ^34^, ^35^] The coupling of HLADH with the water‐forming NOX from *Lactobacillus pentosus* for *in‐situ* recycling of the oxidized NAD^+^ cofactor has shown to even enhance the overall reaction performance.^[31]^ Then, the (*R*)‐selective ADH from *Lactobacillus brevis* (LbADH) was chosen as the second oxidative enzyme, since it shows both excellent enantioselectivity and broad substrate scope.[^3^, ^36^, ^37^] However, LbADH is NADP^+^ dependent, and a highly efficient NADPH oxidase (TPNOX), engineered from *Lactobacillus brevis* NOX was selected as recycling partner.^[38]^ For the amination step, we explored the suitability of the wild‐type (*S*)‐selective TA from *Halomonas elongata* (HEWT),[^39^, ^40^, ^41^] and the (*R*)‐selective thermotolerant TA from *Thermomyces stellatus* (TsRTA).[^42^, ^43^] Both, HEWT and TsRTA accept isopropylamine (IPA) as amino donor resulting in high molar conversions (m.c.).[^31^, ^42^] This one‐pot enzymatic cascade was applied to the asymmetric synthesis of 4‐phenylbutan‐2‐amine from the corresponding racemic alcohol (Scheme 1).

**Scheme 1:**
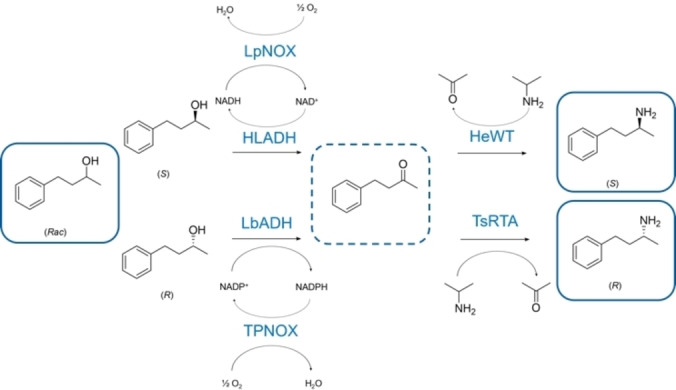
One‐pot enzyme cascade catalysing the conversion of racemic 4‐phenyl‐2‐butanol into either (*S*)‐ or (*R*)‐enantiomers of the corresponding amine with *in‐situ* cofactor recycling.

At 10 mM substrate concentration, the molar conversion (m.c.) of racemic 4‐phenyl‐2‐butanol to either (*S*)‐ or (*R*)‐enantiomer of the amine were modest but encouraging: 49 % and 24 %, respectively, with good to excellent enantiomeric excess (*ee*) (71 % (*S*) and >99 % (*R*)). The m.c. were lower at a higher substrate concentration (50 mM) with both, HEWT and TsRTA (Table 1). In addition, it was confirmed that both ADHs catalyse the oxidation of this racemic secondary alcohol with excellent enantioselectivities (Table S1).

**Table 1:** Bioamination of racemic 4‐phenyl‐2‐butanol in batch reactions catalysed by soluble HLADH (0.5 mg mL^−1^), LbADH (0.5 mg mL^−1^), LpNOX (0.25 mg mL^−1^), TPNOX (0.25 mg mL^−1^), and either HEWT (0.5 mg mL^−1^) or TsRTA (0.5 mg mL^−1^), to synthesize either (*S*)‐ (with HEWT) or (*R*)‐ 4‐phenylbutan‐2‐amine (with TsRTA). Reaction conditions: 10 mM or 50 mM racemic 4‐phenyl‐2‐butanol in phosphate buffer (20 mM, pH 8), 0.1 eq. NAD^+^, 0.1 eq. NADP^+^, 2 eq. IPA, 10 mM MgCl_2_, 1 mM flavin adenine dinucleotide (FAD), and 0.1 mM pyridoxal 5’‐phosphate (PLP). T=30 °C. Reaction volume=1 mL. Mean values of triplicate reactions.

TA	Substrate	Product distribution [%]^[a]^			m.c.	*ee*
	[mM]	Alcohol	Ketone	Amine	[%]^[a]^	[%]^[b]^
HEWT	10	0	50	50	49	71 (*S*)
TsRTA	10	1	73	27	24	>99 (*R*)
HEWT	50	16	48	35	28	88 (*S*)
TsRTA	50	10	80	9	2	>99 (*R*)

[a] Molar conversion (m.c.) determined by HPLC. [b] Enantiomeric excess (*ee*) determined by GC‐FID.

According to the observed product distribution at different time points (Figure S1), the alcohol was oxidised at a faster rate than the following amination step. Therefore, the amino donor system in the synthesis of both, (*S*)‐ and (*R*)‐4‐phenylbutan‐2‐amine, was further optimized. In the application of HEWT to the biocatalytic amination of 4‐phenyl‐2‐butanone, the use of 10 equivalents (eq.) of IPA rather than 2 eq., and a higher enzyme concentration (1 mg mL^−1^), increased the m.c. from 54 % to 75 % at 10 mM scale, and from 42 % to 66 % at 50 mM scale (Table 2).

**Table 2:** Bioamination of 4‐phenyl‐2‐butanone in batch reactions catalysed by soluble HEWT (1 mg mL^−1^) to synthesize (*S*)‐4‐phenylbutan‐2‐amine. Reaction conditions: 10 mM or 50 mM 4‐phenyl‐2‐butanone in phosphate buffer (20 mM, pH 8), 2–10 eq. IPA, and 0.1 mM PLP. T=30 °C. Reaction volume=1 mL. Mean values of triplicate reactions.

Substrate [mM]	IPA [eq.]	m.c. [%]^[a]^
10	2	54
10	5	68
10	10	75
50	2	42
50	5	64
50	10	66

[a] Molar conversion (m.c.) determined by HPLC.

In parallel, for the synthesis of (*R*)‐4‐phenylbutan‐2‐amine catalysed by TsRTA, the same conditions, pushed the m.c. to 53 % at 10 mM scale, while at 50 mM substrate concentration, the highest m.c., 10 %, was obtained with 5 eq. of IPA (Table 3).

**Table 3:** Bioamination of 4‐phenyl‐2‐butanone in batch reactions catalysed by soluble TsRTA (1 mg mL^−1^) to synthesize (*R*)‐ 4‐phenylbutan‐2‐amine. Reaction conditions: 10 mM or 50 mM 4‐phenyl‐2‐butanone in phosphate buffer (20 mM, pH 8), 2–10 eq. IPA or 1–2 eq. (*R*)‐methylbenzylamine (RMBA), and 0.1 mM PLP. T=30 °C. Reaction volume=1 mL. Mean values of triplicate reactions.

Substrate [mM]	Amino donor [eq.]	m.c. [%]^[a]^
10	IPA (2)	33
10	IPA (5)	45
10	IPA (10)	53
10	RMBA (1)	20
10	RMBA (2)	10
50	IPA (2)	9
50	IPA (5)	10
50	IPA (10)	8
50	RMBA (1)	0
50	RMBA (2)	0

[a] Molar conversion (m.c.) determined by HPLC.

Despite these improvements, the m.c. in the TsRTA catalysed reaction was still low for the implementation of this enzymatic cascade for the synthesis of the (*R*)‐amine and further optimization of the concentration of TsRTA was carried out. At 5 mg mL^−1^ of biocatalyst, 58 % and 20 % m.c. were achieved at 10 mM and 50 mM scales, respectively (Table 4).

**Table 4:** Bioamination of 4‐phenyl‐2‐butanone in batch reactions catalysed by soluble TsRTA (1 ‐ 5 mg mL^−1^) to synthesize (*R*)‐ 4‐phenylbutan‐2‐amine. Reaction conditions: 10 mM or 50 mM 4‐phenyl‐2‐butanone in phosphate buffer (20 mM, pH 8), 5 eq. (for 10 mM substrate concentration) or 10 eq. IPA (for 50 mM substrate concentration), and 0.1 mM PLP. T=30 °C. Reaction volume=1 mL. Mean values of triplicate reactions.

Substrate [mM]	TsRTA concentration	m.c. [%]^[a]^
10	1 mg mL^−1^	53
10	2 mg mL^−1^	58
10	5 mg mL^−1^	58
50	1 mg mL^−1^	10
50	2 mg mL^−1^	13
50	5 mg mL^−1^	20

[a] Molar conversion (m.c.) determined by HPLC.

The enzymatic cascade consisting of HLADH‐LbADH‐LpNOX‐TPNOX and either HEWT or TsRTA, with optimised reaction conditions in the amination step, was then applied to the synthesis of the (*S*)‐ or (*R*)‐enantiomers of the target amine (Table 5). In the synthesis of (*R*)‐4‐phenylbutan‐2‐amine from racemic alcohol, 61 % and 35 % m.c. were respectively achieved at 10 mM and 50 mM substrate concentrations, with excellent enantioselectivity (>99 % *ee*) which nicely compares with the system developed by Mutti and co‐workers which established an enzymatic cascade formed by two enantiocomplementary ADHs, AmDH, NADPH oxidase, catalase and formate dehydrogenase, albeit such system yielded complete conversion of the same substrate.^[44]^ The lower m.c. obtained in the present work might be due to operational stability issues of TsRTA, but engineering of this enzyme was recently reported to yield a more stable variant.^[42]^

**Table 5:** Bioamination of racemic 4‐phenyl‐2‐butanol in batch reactions catalysed by soluble HLADH (0.5 mg mL^−1^), LbADH (0.5 mg mL^−1^), LpNOX (0.25 mg mL^−1^), TPNOX (0.25 mg mL^−1^), and either HEWT (1 mg mL^−1^) or TsRTA (5 mg mL^−1^), to synthesize either (*S*)‐ (with HEWT) or (*R*)‐4‐phenylbutan‐2‐amine (with TsRTA). Reaction conditions: 10 mM or 50 mM racemic 4‐phenyl‐2‐butanol in phosphate buffer (20 mM, pH 8), 0.1 eq. NAD^+^, 0.1 eq. NADP^+^, 10 eq. (with HEWT and with TSRTA at 10 mM substrate concentration) or 5 eq. IPA (with TSRTA at 50 mM substrate concentration), 10 mM MgCl_2_, 1 mM FAD, and 0.1 mM PLP. T=30 °C. Reaction volume=1 mL. Mean values of triplicate reactions.

TA	Substrate	Product distribution (%)^[a]^			m.c.	*ee*
	[mM]	Alcohol	Ketone	Amine	[%]^[a]^	[%]^[b]^
HEWT	10	0	18	82	66	44 (*S*)
TsRTA	10	0	28	72	61	>99 (*R*)
HEWT	50	0	36	64	67	73 (*S*)
TsRTA	50	0	55	45	35	>99 (*R*)

[a] Molar conversion (m.c.) determined by HPLC. [b] Enantiomeric excess (*ee*) determined by GC‐FID.

The synthesis of (*S*)‐4‐phenylbutan‐2‐amine at 50 mM was obtained with very good m.c. (67 %), and this value compares to a previously reported cascade which, however, required the optically pure (*S*)‐alcohol and generated both alanine and lactate.^[25]^ In addition, Li and co‐workers developed a novel simple system containing only ADH, TA and IPA for the amination of racemic alcohol to produce (*S*)‐ amine.^[28]^ However, they applied this enzymatic cascade only *in‐vivo*, and the whole‐cell yielded the (*S*)‐amine with 88 % m.c. and >99 % *ee*.

In this work, we described a cell‐free biocatalytic system for the tunable conversion of racemic 4‐phenyl‐2‐butanol into either (*S*)‐ or (*R*)‐enantiomers of 4‐phenylbutan‐2‐amine. These high value enantiomers of the aromatic amine were synthesized in one‐pot with good (73 % (*S*)) and excellent (>99 % (*R*)) enantioselectivities from the corresponding racemic alcohol. To improve further on the applicability of this one‐pot enzymatic cascade to the synthesis of (*R*)‐4‐phenylbutan‐2‐amine, engineering TsRTA for improved operational stability might be an interesting approach. In addition, this work represents the first example of synthesis of (*S*)‐4‐phenylbutan‐2‐amine from the racemic alcohol using isolated enzymes with good conversion and the highest *ee* reported up to date. Thus, this work provides a biocatalytic system as an alternative for the direct synthesis of chiral amines from racemic alcohols and contributes to further expand the toolbox of biocatalysis for its application to the pharmaceutical industry. As an outlook, the immobilisation of the biocatalysts and the translation of the reaction to a continuous flow set up would make the process even more efficient.

## Conflict of interest

The authors declare no conflict of interest.

## Supporting information

As a service to our authors and readers, this journal provides supporting information supplied by the authors. Such materials are peer reviewed and may be re‐organized for online delivery, but are not copy‐edited or typeset. Technical support issues arising from supporting information (other than missing files) should be addressed to the authors.

Supporting Information

## Data Availability

The data that support the findings of this study are available from the corresponding author upon reasonable request.
