# Supplementary material for: Stereo‐Divergent Enzyme Cascades to Convert Racemic 4‐Phenyl‐2‐Butanol into either (S)‐ or (R)‐Corresponding Chiral Amine
Source: Chembiochem. 2022 Mar 3;23(8):e202200108. doi: 10.1002/cbic.202200108 (PMC9313814; doi:10.1002/cbic.202200108)
Supplement: Supplementary file 1 — Supporting Information [file CBIC-23-0-s001.pdf]

ChemBioChem

Supporting Information

Stereo-Divergent Enzyme Cascades to Convert Racemic 4-Phenyl-2-Butanol into either (*S*)- or (*R*)-Corresponding Chiral Amine

Maria Romero-Fernandez and Francesca Paradisi*

Materials

All chemicals and reagents used for this work were purchased from Sigma-Aldrich unless specified otherwise. *Escherichia coli* BL21(DE3) Star competent cells and E. coli XL10-Gold® Ultracompetent cells were respectively purchased from Themo Fisher Scientific and Agilent. Q5® High-Fidelity DNA polymerase, BamHI-HF® and EcoRI-HF® restriction enzymes, and T4 DNA ligase were acquired from New England Biolabs. QIAquick PCR Purification Kit was purchased from Qiagen. GeneJET PCR Purification Kit was purchased from Thermo Fisher Scientific. Anydrotetracycline was purchased from Cayman Chemical Company. StrepTrap HP 5 mL and HisTrap HP 1 mL columns were acquired from GE Healthcare. Nicotinamide adenine dinucleotide (phosphate) oxidised ($NAD(P)^+$) and reduced (NAD(P)H) forms were purchased from Apollo Scientific. Racemic 4-phenyl-2-butanol and *(S)*- 4-phenylbutan-2-amine was purchased from Acros Organics. 4-phenyl-2-butanone and alpha-toluenesulfonyl fluoride (PMSF) were acquired from Alfa Aesar. IPTG was purchased from VWR Life Science and $MgCl_2$ was purchased from VWR Chemicals.

Methods

Cloning of TPNOX

The plasmid harbouring TPNOX (pUC57_TPNOX) was acquired from Addgene (Plasmid catalogue number: 87853). The gene was amplified by PCR with Q5® High-Fidelity DNA polymerase with the oligonucleotide primers BamHI_TPNOX_fwd (5′- ATTGGATCCAATGAAGGTCACCGT -3′) and EcoRI_TPNOX_rv (5′-ATGGAATTCTTACTTGTCATC-3′). The primers were designed to incorporate BamHI and EcoRI restriction sites, respectively (underlined). The manufacturer's protocol for Q5® High-Fidelity DNA polymerase PCR in 50 µL reaction was used. PCR was carried out with 10 µL 5× Q5® High-Fidelity DNA polymerase buffer; 2.5 µL 10 mM BamHI_TPNOX_fwd primer; 2.5 µL 10 mM EcoRI_TPNOX_rv primer; 1 µL 10 mM dNTPs; 31.5 µL nuclease-free water; 2 µL pUC57_TPNOX; and 0.5 µL Q5® High-Fidelity DNA polymerase. PCR conditions were 1 min of at 95 °C and 35 cycles of 15 seconds min denaturation at 95 °C, 15 seconds min annealing at 60 °C and 2 min extension at 72 °C. The 35 cycles were followed by 7 min of final elongation at 72 °C. The amplified DNA was purified with a QIAquick PCR Purification Kit. The purified PCR product was digested with BamHI-HF® and EcoRI-HF® for 2 h at 37 °C. The digested gene was gel-extracted using a GeneJET PCR Purification Kit and cloned into the pMP89b[1] vector, digested with the same restriction enzymes. Ligation was developed with T4 DNA ligase following the manufacturer's guidelines and the resulting product was used to transform E. coli XL10-Gold® Ultracompetent cells. The construct was verified by sequencing and the obtained plasmid was named pMP89b_TPNOX.

Enzyme expression

The previously reported expression protocols of HLADH,[2] LpNOX,[2] HEWT[1] and TsRTA[3] were followed. The plasmid harbouring LbADH (pASK-IBA5plus-LbADH), kindly donated by Prof. Kroutil, was transformed into *E. coli* BL21(DE3) Star. 1 L flasks containing 300 mL of Terrific Broth media, supplemented with 0.3 mM $MgCl_2$, were inoculated with 10 mL of an overnight culture in LB and left to grow at 37 °C and 180 rpm until OD600 reached 0.6–0.7. At that point, the expression was induced with 0.4 µM anhydrotetracycline (final concentration) and the cultures were left to grow for 16 h at 20 °C and 180 rpm. For TPNOX, a single colony of *E. coli* BL21 (DE3) Star cells previously transformed with the plasmid pMP89b-TPNOX was inoculated in 10 mL overnight culture in LB. 1 L flasks containing 300 mL of Terrific Broth media were inoculated with 10 mL of overnight culture and left to grow at 37 °C and 180 rpm until OD600 reached 0.4–0.5. At that point, the expression was induced with 0.1 mM IPTG (final concentration) and the cultures were left to grow for 16 h at 15 °C and 180 rpm. The cells were collected by centrifugation at 4500 rpm and stored at −20 °C until further use.

Enzyme purification

The previously reported purification protocols of HLADH,[2] LpNOX,[2] HEWT[2] and TsRTA[3] enzymes were followed. The cells containing expressed LbADH enzyme were resuspended in 20 mM sodium phosphate buffer, 0.28 M NaCl, and 6 mM KCl 1 mM $MgCl_2$ pH 7.4. Cells were disrupted by sonication with pulses of 5 seconds on and 5 seconds off at 60% amplitude for 12 min. The insoluble fraction was separated by centrifugation at 14 500 rpm for 60 min. The supernatant was filtered (0.45 µm) and loaded into a StrepTrap HP 5 mL column using an ÄKTA™ start FPLC (GE Healthcare). The protein was eluted with 20 mM sodium phosphate buffer, 0.28 M NaCl, 6 mM KCl, 1 mM $MgCl_2$ and 2.5 mM D-desthiobiotin pH 7.4. LbADH was dialysed twice against 100 mM potassium phosphate buffer and 1 mM $MgCl_2$ pH 7 at 4 °C. Cells containing expressed TPNOX enzyme were resuspended in 50 mM sodium phosphate buffer, 0.5 M NaCl, 20 mM imidazole, 0.1 mM flavin adenine dinucleotide (FAD), and 0.1 mM alpha-toluenesulfonyl fluoride (PMSF) pH 8. After cell disruption and insoluble fraction separation under the same conditions as before, the filtered supernatant was loaded into a HisTrap HP 1 mL column using the ÄKTA™ start FPLC. The protein was eluted with 50 mM sodium phosphate buffer, 0.5 M NaCl, 300 mM imidazole, 0.1 mM FAD pH 8, and dialysed twice against 50 mM sodium phosphate buffer 0.01 mM FAD pH 7.5 at 4 °C.

Enzymatic activity assays

All enzyme assays were performed in triplicate in 96-well microplates using an Epoch 2 Microplate Spectrophotometer (Biotek). For HLADH,[2] LpNOX,[2] HeWT,[2] and TsRTA[3] the activity assays were performed as previously indicated. LbADH activity assay was developed following the formation of NADPH at 340 nm using 40 mM 1-phenylethanol, 16% DMSO, 1 mM $NADP^+$, and 1 mM $MgCl_2$ in 50 mM phosphate buffer at 25 °C and pH 8. TPNOX activity assay was performed following the depletion of NADPH at 340 nm using 0.25 mM NADH in 50 mM phosphate buffer at 25 °C and pH 8. One unit of enzymatic activity was defined as the oxidation of 1 µmol of cofactor per minute.

Determination of protein concentration

HLADH, HEWT, TsRTA and LbADH protein concentration were determined by UV absorption at 280 nm using an Epoch Take3 Micro-Volume Plate. The extinction coefficients 22 460 M^{-1} cm^{-1}, 62 340 M^{-1} cm^{-1}, 53 860 M^{-1} cm^{-1} and 25 440 M^{-1} cm^{-1}, at 280 nm, measured in water, were respectively estimated for HLADH, HEWT, TsRTA and LbADH using the ExPASy ProtParam tool, accessible from the ExPASy website (http://www.expasy.ch). LpNOX and TPNOX enzymes concentrations were determined using a Bradford assay with bovine serum albumin (BSA) as the standard.

Batch reaction of biocatalytic amination of racemic 4-phenyl-2-butanol

Batch reactions with pure soluble enzymes were performed at 30 °C in 1 mL of reaction mixture containing 10 mM or 50 mM racemic 4-phenyl-2-butanol, 0.1 eq. (1 mM or 5 mM) NAD^+, 0.1 eq. (1 mM or 5 mM) $NADP^+$, 2 – 10 eq. (20 – 500 mM) IPA, 0.1 mM PLP, 1 mM FAD, 10 mM $MgCl_2$, 20 mM potassium phosphate buffer pH 8, HLADH (0.5 mg mL^{-1}), LbADH (0.5 mg mL^{-1}), LpNOX (0.25 mg mL^{-1}), TPNOX (0.25 mg mL^{-1}), and either HEWT (0.5 – 1 mg mL^{-1}) or TsRTA (0.5 – 5 mg mL^{-1}). The reactions were monitored by HPLC, and enantioselectivities were determined by GC-FID, as described in the *Analytical methods* section.

Batch reaction of biocatalytic amination of 4-phenyl-2-butanone

Batch reactions with pure soluble enzymes were performed at 30 °C in 1 mL of reaction mixture containing 10 mM or 50 mM 4-phenyl-2-butanone, 2 – 10 eq. (20 – 500 mM) IPA or 1 – 2 eq. (10 – 100 mM) *(R)*-methylbenzylamine (RMBA) (with TsRTA), 0.1 mM PLP, and either HEWT (1 mg mL^{-1}) or TsRTA (1 – 5 mg mL^{-1}). The reactions were monitored by HPLC, as described in the *Analytical methods* section.

Analytical methods

Biocatalytic reactions were monitored by HPLC as follows. 100 µL of sample with a maximum concentration of substrate (10 mM) were added to 450 µL acetonitrile and 450 µL 0.2% HCl. The samples were analysed by HPLC on a Waters XBridge C18 column (3.5µm, 2.1 × 150mm), measuring at 210 nm, with a gradient method of phase A (0.1% TFA in water) and B (0.1% TFA in acetonitrile). Gradient: 0 min 95% A 5% B; 1 min 95% A 5% B; 5min 5% A 95% B; 5.10 min 0% A 100% B; 6.60 min 0% A 100% B; 7min 95% A 5% B; 10 min 95% A 5% B. Injection volume 2 µL, at 45º C with a flow rate of 0.8 mL min^{-1}. The retention times of the different substances were: 4-phenyl-2-butanol 4.21 min, 4-phenyl-2-butanone 4.33 min, and 4-phenylbutan-2-amine 3.4 min. Molar conversions (m. c.) were calculated through a standard curve of the product.

Enantioselectivity was determined by GC-FID as follows. Samples were added to 100 µL of 5 M sodium hydroxide and extracted into 2 × 500 µL ethyl acetate. Extracted samples were derivatized with 20 µL triethylamine and 20 µL acetic anhydride. Analysis was developed by GC-FID (Thermo Scientific™ Trace™ 1310 GC) on an Agilent CHIRASIL-DEX CB (25 m x 0.25 mm x 0.25 µm) column: 0 min 40 °C, 1 min 40 °C, 4 min 100 °C, 5 min 100 °C, 15 min 110 °C, 16 min 110 °C, 17.8 min 200 °C, 22.8 min 200 °C. Injector temperature 230 °C, split ratios 1:3 to 1:100, with a flow rate of 1.7 mL min^{-1}, FID temperature 250 °C, and injection volume 1 µL. Helium was used as carrier gas. The retention times of the different substances were: *(S)*-4-phenyl-2-butanol 9.683 min; *(R)*-4-phenyl-2-butanol 9.745 min; *(S)*-4-phenylbutan-2-amine 14.562 min; and *(R)*- 4-phenylbutan-2-amine 14.610 min. Enantiomeric excess (*ee*) was calculated as the ratio of one enantiomer to the sum of both enantiomers.

Supporting Figures

Biocatalytic amination reactions of a single enantiomer of racemic 4-phenyl-2-butanol

Batch reactions with pure soluble enzymes were performed at 30 °C for 48 hours in 1 mL of reaction mixture containing 10 mM or 50 mM racemic 4-phenyl-2-butanol, either 0.1 eq. (1 mM or 5 mM) NAD^+ or 0.1 eq. (1 mM or 5 mM) $NADP^+$, 2 eq. (20 mM or 100 mM) IPA, 0.1 mM PLP, 1 mM FAD, 10 mM $MgCl_2$, 20 mM potassium phosphate buffer pH 8, either HLADH (0.5 mg mL^{-1}) or LbADH (0.5 mg mL^{-1}), either LpNOX (0.25 mg mL^{-1}) or TPNOX (0.25 mg mL^{-1}), and either HEWT (0.5 mg mL^{-1}) or TsRTA (0.5 mg mL^{-1}). Enantiomeric excess (*ee*) of alcohol and amine were determined by GC-FID, as described in the *Analytical methods* section.

Table S1. Enantiomeric excess of alcohol and amine at 48 hours of bioamination reactions of a single enantiomer of racemic 4-phenyl-2-butanol, catalysed by either HLADH + LpNOX or LbADH + TPNOX, and either HEWT or TsRTA. Reaction conditions: 10 mM or 50 mM racemic 4-phenyl-2-butanol in phosphate buffer (20 mM, pH 8), 0.1 eq. NAD^+, 0.1 eq. $NADP^+$, 2 eq. IPA, 10 mM $MgCl_2$, 1 mM FAD, 0.1 mM PLP, HLADH (0.5 mg mL^{-1}), LbADH (0.5 mg mL^{-1}), LpNOX (0.25 mg mL^{-1}), TPNOX (0.25 mg mL^{-1}), HEWT (0.5 mg mL^{-1}) and TsRTA (0.5 mg mL^{-1}). T = 30 °C. Reaction volume = 1 mL. Mean values of triplicate reactions.

Substrate concentration (mM)	ADH + NOX	TA	Alcohol (*ee*)	Amine (*ee*)
10	HLADH + LpNOX	HEWT	>99% *(R)*	77% *(S)*
	LbADH + TPNOX	HEWT	>99 *(S)*	81% (S)
	HLADH + LpNOX	TsRTA	>99% *(R)*	>99 *(R)*
	LbADH + TPNOX	TsRTA	>99 *(S)*	>99 *(R)*
50	HLADH + LpNOX	HEWT	>99% *(R)*	90% *(S)*
	LbADH + TPNOX	HEWT	93% *(S)*	86% *(S)*
	HLADH + LpNOX	TsRTA	>99% *(R)*	>99 *(R)*
	LbADH + TPNOX	TsRTA	94% *(S)*	>99 *(R)*

Product distribution over time of biocatalytic amination reactions of racemic 4-phenyl-2-butanol

Batch reactions with pure soluble enzymes were performed at 30 °C in 1 mL of reaction mixture containing 10 mM or 50 mM racemic 4-phenyl-2-butanol, 0.1 eq. (1 mM or 5 mM) NAD^+, 0.1 eq. (1 mM or 5 mM) $NADP^+$, 2 eq. (20 or 100 mM) IPA, 0.1 mM PLP, 1 mM FAD, 10 mM $MgCl_2$, 20 mM potassium phosphate buffer pH 8, HLADH (0.5 mg mL^{-1}), LbADH (0.5 mg mL^{-1}), LpNOX (0.25 mg mL^{-1}), TPNOX (0.25 mg mL^{-1}), and either HEWT (0.5 mg mL^{-1}) or TsRTA (0.5 mg mL^{-1}). The reactions were monitored at different time points by HPLC, as described in the *Analytical methods* section.

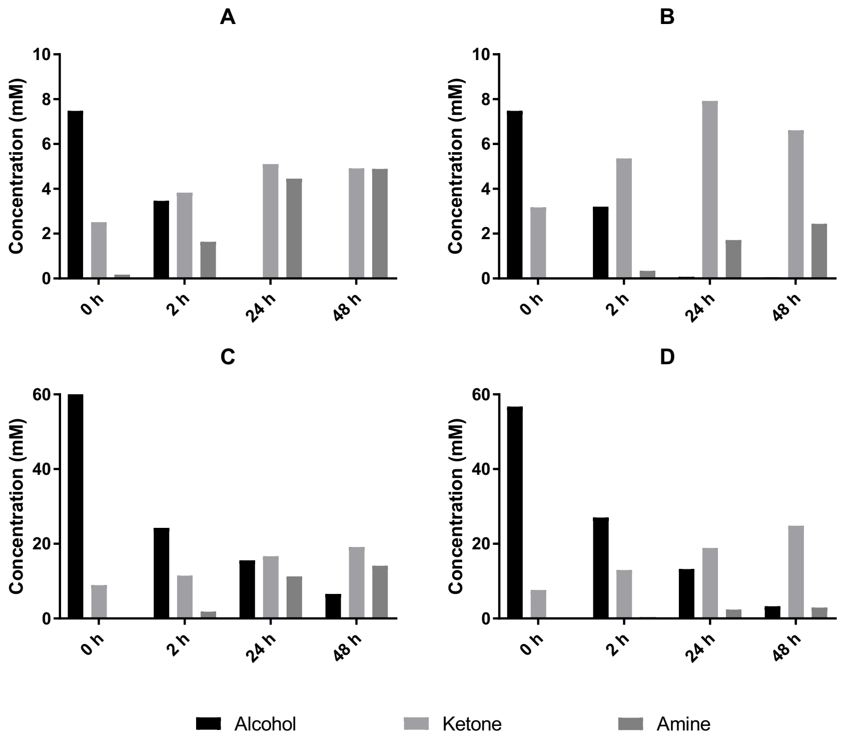

Fig. S1. Product distribution over time of bioamination reactions of 10 mM (A and B) or 50 mM (C and D) racemic 4-phenyl-2-butanol in batch catalysed by soluble HLADH, LbADH, LpNOX, TPNOX, and either HEWT (A and C) or TsRTA (B and D). Reaction conditions: 10 mM or 50 mM racemic 4-phenyl-2-butanol in phosphate buffer (20 mM, pH 8), 0.1 eq. NAD^+, 0.1 eq. $NADP^+$, 2 eq. IPA, 10 mM $MgCl_2$, 1 mM FAD, 0.1 mM PLP, HLADH (0.5 mg mL^{-1}), LbADH (0.5 mg mL^{-1}), LpNOX (0.25 mg mL^{-1}), TPNOX (0.25 mg mL^{-1}), and either HEWT (0.5 mg mL^{-1}) or TsRTA (0.5 mg mL^{-1}). T = 30 °C. Reaction volume = 1 mL. Mean values of triplicate reactions.

Supporting references

[1] L. Cerioli, M. Planchestainer, J. Cassidy, D. Tessaro, F. Paradisi, *J. Mol. Catal. B Enzym.* **2015**, *120*, 141–150.

[2] M. Romero-Fernandez, F. Paradisi, *Green Chem.* **2021**, *23*, 4594–4603.

[3] C. M. Heckmann, L. J. Gourlay, B. Dominguez, F. Paradisi, *Front. Bioeng. Biotechnol.* **2020**, *8*, 707.
